# Multiplatform molecular analysis of vestibular schwannoma reveals two robust subgroups with distinct microenvironment

**DOI:** 10.1007/s11060-022-04221-2

**Published:** 2023-01-26

**Authors:** Alexander P. Landry, Justin Z. Wang, Suganth Suppiah, Gelareh Zadeh

**Affiliations:** Division of Neurosurgery, University of Toronto, Toronto, ON Canada

**Keywords:** Vestibular schwannoma, Acoustic neuroma, Molecular analysis, Immunotherapy, Subgroup analysis, Methylation, Transcriptomics

## Abstract

**Background:**

Vestibular schwannoma (VS) is the most common tumour of the cerebellopontine angle and poses a significant morbidity for patients. While many exhibit benign behaviour, others have a more aggressive nature and pattern of growth. Predicting who will fall into which category consistently remains uncertain. There is a need for a better understanding of the molecular landscape, and important subgroups therein, of this disease.

**Methods:**

We select all vestibular schwannomas from our tumour bank with both methylation and RNA profiling available. Unsupervised clustering methods were used to define two distinct molecular subgroups of VS which were explored using computational techniques including bulk deconvolution analysis, gene pathway enrichment analysis, and drug repurposing analysis. Methylation data from two other cohorts were used to validate our findings, given a paucity of external samples with available multi-omic data.

**Results:**

A total of 75 tumours were analyzed. Consensus clustering and similarity network fusion defined two subgroups (“immunogenic” and “proliferative”) with significant differences in immune, stroma, and tumour cell abundance (p < 0.05). Gene network analysis and computational drug repurposing found critical differences in targets of immune checkpoint inhibition PD-1 and CTLA-4, the MEK pathway, and the epithelial to mesenchymal transition program, suggesting a need for subgroup-specific targeted treatment/trial design in the future.

**Conclusions:**

We leverage computational tools with multi-omic molecular data to define two robust subgroups of vestibular schwannoma with differences in microenvironment and therapeutic vulnerabilities.

**Supplementary Information:**

The online version contains supplementary material available at 10.1007/s11060-022-04221-2.

## Background

Vestibular schwannoma (VS) is the most common tumour of the cerebellopontine angle (CPA) and represents 6–8% of all intracranial tumours [1]. While many are discovered incidentally, symptomatic patients typically present with progressive hearing loss, tinnitus, and in larger tumours, may develop hydrocephalus, symptomatic brainstem compression, headache, and/or cranial nerve dysfunction (e.g. trigeminal pain, and in rare cases facial weakness) [2]. The majority of tumours grow slowly at an average rate of 1–2 mm/year, with up to 75% of tumors showing no radiographic growth after diagnosis [3]. However, a subset of these tumours exhibit more aggressive behaviour with rapid growth and a propensity to recur after treatment with microsurgical resection, stereotactic radiosurgery, or both [4].

Histopathologically, VS is a benign neoplasm arising from Schwann cells of the vestibular nerve. It is known to be associated with the *NF2* gene and it’s product, Merlin, which is associated with several key receptors such as EGFR and several entities within the Ras and Wnt pathways [5]. Several biomarkers associated with tumour growth have been identified including elements of the Merlin pathway, inflammatory signals including NFKB1, COX genes, and macrophages [6], an immune-enriched microenvironment [7], and the *SH3PDX2A-HTRA1* fusion [8], though these have yet to find their way into clinical practice. While there is an ongoing push toward defining CNS tumours by their molecular features (as evidenced by recent changes to WHO tumour classification [9]), WHO continue to define VS as a single entity despite their demonstrated potential for highly heterogeneous behaviour. Similarly, while the VEGF inhibitor bevacizumab has shown promise in reducing tumour cell proliferation and improving hearing in a subset of patients [10], and our lab has previously shown that patients with *SH3PDX2A-HTRA1* fusion positive tumours may respond to inhibition of the MEK-ERK pathway [8], there remain an absence of medical treatments in their standard care. Overall, there is an outstanding need for subgroup discovery, outcome prediction, and targeted therapeutics for patients with vestibular schwannoma.

In this study, we explore the transcriptome and methylome of vestibular schwannomas. We identify subgroups driven by multiplatform molecular profiling. The results add to our knowledge of schwannomas that can inform increasingly personalized care for patients with this challenging disease.

## Methods

### Data collection and pre-processing

This is a retrospective cohort study. Research and Ethics board approval was obtained (18-5820). We selected all vestibular schwannomas from our local tumour bank treated with upfront microsurgical resection that had been sequenced for both gene expression and DNA methylation to form the discovery cohort [8]. Tumours were resected via either the retrosigmoid or translabyrinthine approach depending on tumour morphology, surgeon preference, and preoperative hearing status, and fresh tissue taken intraoperatively for processing. Tumors for the UHN Tumour Bank are sent directly from the operating room, where samples are placed in aliquots marked by location in storage tubes placed in liquid nitrogen vats in the operating room. Each tumor has multiple samples from different regions collected and where possible duplicates or more. DNA and RNA were extracted from fresh frozen tumour tissue using the DNeasy Blood and Tissue Kit (Qiagen, USA), and RNAeasy Mini Kit (Qiagen, USA). DNA was quantified using Qubit dsDNA HS Assay Kit and RNA integrity was evaluated using the Agilent 2100 Bioanalyzer (RNA; Agilent, USA) with only samples that had an RNA Integrity Number (RIN) > 7 selected for subsequent sequencing. Illumina Infinium Methylation EPIC BeadChip Array were used to obtain genome-wide DNA methylation profiles on vestibular schwannomas following bisulfite conversion using the EZ DNA Methylation Kit (Zymo, USA). mRNA libraries were generated using the NEB Ultra II directional mRNA library prep kit. Libraries were sequenced on the Illumina HiSeq to obtain approximately 70 million reads per sample. FastQ files were processed and aligned to human reference genome (GRCh38) using STAR (v2.6.0a). Raw gene expression counts were normalized by counts-per-million. DNA methylation data was processed using the minfi package [11], quantile normalized, and beta values were used for subsequent analysis. Additional tumours from the same cohort (combined samples from Toronto and MD Anderson, Texas) [8] which were sequenced only for methylation were used as an internal validation cohort (validation cohort 1). Notably, we found a significant batch effect between sequencing groups and this was corrected using ComBat [12], a commonly used Bayesian technique. We also use available data from the DKFZ reference cohort database [13],[14] (methylation data only) as an external validation cohort (validation cohort 2). DNA was extracted from formalin fixed paraffin embedded tumour tissue using the automated Maxwell system (Promega, Madison, WI, USA) and Illumina Infinium HumanMethylation450 used for methylation profiling [14]. Subsequent computational analysis proceeded as in the discovery and internal validation cohorts. Notably, analysis in our study was done using the open-source platform R, version 3.6.3 [15].

### Subgroup discovery with molecular clustering

To identify homogeneous molecular subgroups, we first selected the 5000 most variable genes and methylation probes to be used in subsequent clustering analysis. We used three distinct clustering methods (similarity network fusion [SNF] [16] and cluster of cluster analysis [COCA] [17] using both hierarchical and k-means clustering) to identify subgroups; final group membership was determined by consensus. Similarity network fusion (SNF) applies spectral clustering to each input data modality (mRNA and methylation) which are subsequently “fused” using a mixing function to generate a single heatmap. Optimal number of clusters is determined by minimizing the eigengap. Cluster of cluster analysis generalizes clustering methods (hierarchical, k-means, etc.) across multiple platforms such that the most stable overall cluster assignment is selected. Once subgroups were identified using all three methods, any disagreement in group assignment was solved using majority. T-SNE [18] analysis was used to visualize groupings using both methylome and transcriptome as input data. Expression of each gene/methylation probe was compared between resultant subgroups using a t-test.

To generate similar subgroups in the validation cohorts, we created a methylation signature to define subgroups in the validation cohort. This was done by selecting the 100 most differentially expressed probes between subgroups (ordered by q value) and using the mean value of up- and down-regulated probes as regressors in a logistic regression model. 5-fold cross validation yielded 100% prediction accuracy and the regression model was therefore applied to both validation cohorts to generate subgroup labels.

### Tumour microenvironment analysis

We applied the ESTIMATE tool [19] to gene expression data from the discovery cohort to determine the approximate relative populations of stromal, immune, and neoplastic cells within the microenvironment of each tumour. Briefly, this method uses bulk deconvolution to compare input gene expression data to previously established transcriptomic “signatures” of immune and stromal cell populations. These were established through differential gene expression analysis over a large pan-cancer analysis using multiple databases resulting in a stromal signature containing 141 genes and an immune signature containing 141 genes. Tumour purity (proportion of neoplastic cells) was validated against genomic data in the development of the tool as well. These characteristics were compared between subgroups using a t-test.

To validate differences in microenvironment, we sought methylation probes which best correlated with immune, stromal, and purity scores in the discovery cohort since these scores are generated from transcriptomic input. Mean beta values of the 10 most correlated probes to each parameter was selected to be a marker of these microenvironment parameters. The relative proportion of each cell type in the validation cohort was therefore estimated as the difference in these markers (between subgroups) in the validation cohort.

In additional to the transcriptomic-based ESTIMATE, we calculated the methylation-based LUMP (leukocytes unmethylation to infer tumour purity) scores on all tumours. This method uses a previously validated methylation signature to infer tumour purity [20]. Unfortunately, to our knowledge, a complementary methylation-based method for inferring immune and stromal populations using methylation data alone doesn’t exist, necessitating the indirect approach outlined above.

### Gene pathway analysis

We used *Gene Set Enrichment Analysis (GSEA)* [21],[22], to identify gene networks associated with targets of interest. Expression of each constituent gene was compared between subgroup, and network analysis was carried out using Cytoscape [23]. In this latter network analysis, genes which are differentially expressed between subgroups (p < 0.05) and which co-express with other genes from the same network (Pearson p < 0.05) were included.

### Drug repurposing analysis

We applied the openly available L1000 connectivity map [24] in order to identify subgroup-specific drug candidates for VS. Briefly, this method is built upon a repository of transcriptomic perturbations associated with the effects of small molecules on cell lines. By comparing these perturbations with differential gene expression from a disease state, drugs with the potential to reverse a diseased phenotype can be identified. This has a significant benefit over other methods of drug discovery given that the queried compounds are already approved and in use for other conditions, thereby eliminating the need for lengthy testing and approval processes. The top 100 upregulated and downregulated genes between subgroups were used as input.

## Results

### Overview of study cohort

The discovery cohort included 16 vestibular schwannomas treated with primary microsurgery and profiled for bulk transcriptomic and DNA methylation data. Mean age (SD) was 40.3 (13.6), and 10/16 patients were male. The mean follow-up time was 61.8 months (SD 38.8 months, range 12–110) of the 10 patients with available data. Of these ten patients, none had preoperative facial palsy or hydrocephalus, and mean tumour size was 26.7 mm (SD 9.4 mm). One patient had postoperative progression at 105 months treated with radiotherapy and another underwent radiotherapy 17 months postoperatively due a large residual and bothersome trigeminal symptoms. Twelve patients had NF2 point mutations, six had chromosome 22 loss, and three were NF2 intact. Our internal validation cohort included 48 tumours with methylation profiling. Mean age (SD) was 50.0 (11.6) and 20/48 patients were male. 35 patients had chromosome 22 loss. A second validation cohort, using publicly available DKFZ methylation data on a repository of CNS tumours [13] included 11 vestibular schwannomas. In this cohort, the mean (SD) age was 21.6 (14.6) years and 6/11 patients were male. Further clinical annotation is limited in this cohort, though 3/7 patients with documented status are noted to have neurofibromatosis type 2^14^.

### Subgroup discovery

**Fig. 1:**
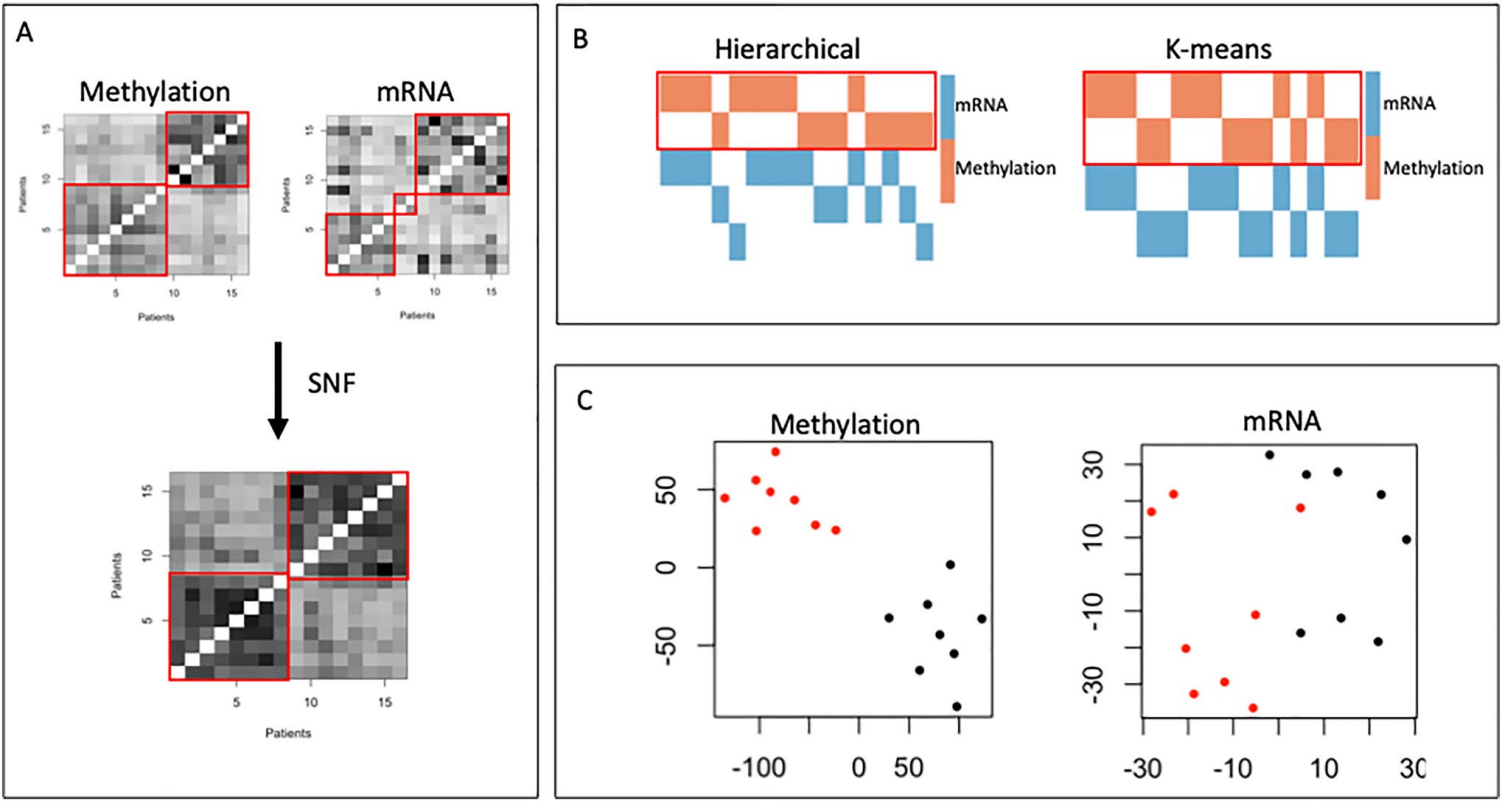
Methylation and mRNA data reveal two robust molecular subtypes of vestibular schwannoma. **A**: Similarity network fusion. Spectral clustering of methylation data reveals two groups from the methylation data and three groups from the mRNA data with the eigengap minimization method. Combining these with similarity network fusion (SNF) yields two equal-sized groups. **B**: Consensus clustering with hierarchical (left) and K-means clustering (right). Methylation cluster assignments are coloured orange and mRNA cluster assignment in blue. The cluster of cluster assignment is denoted by a red box. **C**: t-SNE analysis of tumour methylome and transcriptome, coloured by subgroup (group 1 in black and group 2 in red)

Each clustering method generated two subgroups with eight tumours each (Fig. 1). SNF and COCA-km generated identical group assignments and were taken as consensus. Interestingly, COCA analysis generated groups which were equivalent to the clustering achieved by methylation alone, suggesting that DNA methylation drives clustering for VS.

### Subgroup comparison

**Fig. 2:**
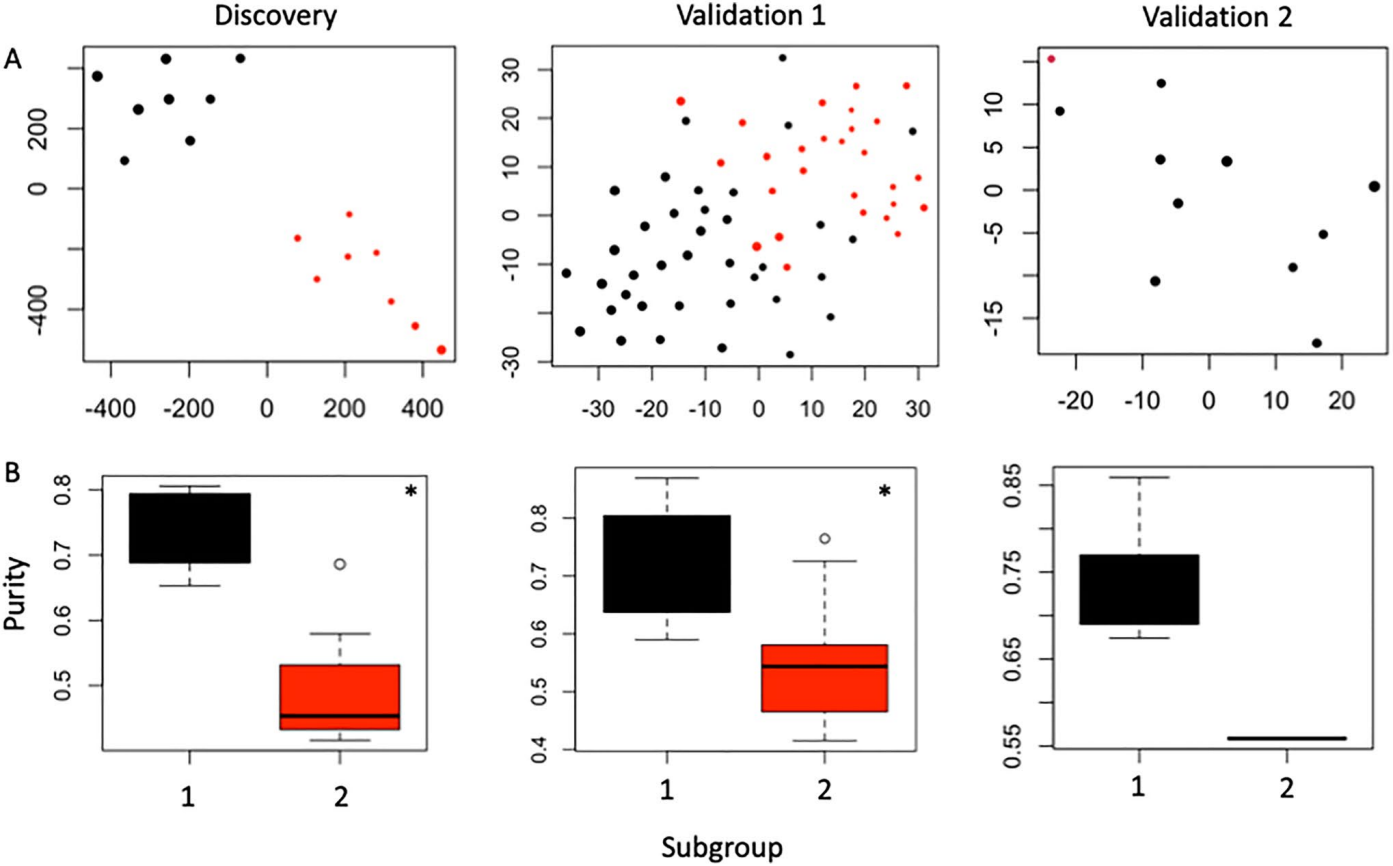
Validation of molecular subgroups on external cohorts. **A**: t-SNE plots of individual tumours by methylation profile (top 5000 most variable probes). Colour depicts subgroup membership (black is group 1 and red is group 2) and size depicts relative LUMP score. **B**: Boxplots comparing mean LUMP score by subgroup in each cohort. *p < 0.05

Our final consensus subgroups (derived equivalently from both SNF and COCA-km) consisted of eight tumours each (discovery cohort). There was no significant difference in age, sex, or tumour size between groups (p > 0.05). In group 1, no patients had postoperative progression or further treatment whereas 2 patients with documented follow up in group 2 did. In the internal validation cohort, 33 tumours were assigned as group 1 and 15 as group 2; in the external validation cohort 10 tumours were assigned group 1 and one was assigned group 2. There are no significant differences in age or sex (p > 0.05) by subgroup in the two validation cohorts (Fig. 2 A).

**Fig. 3:**
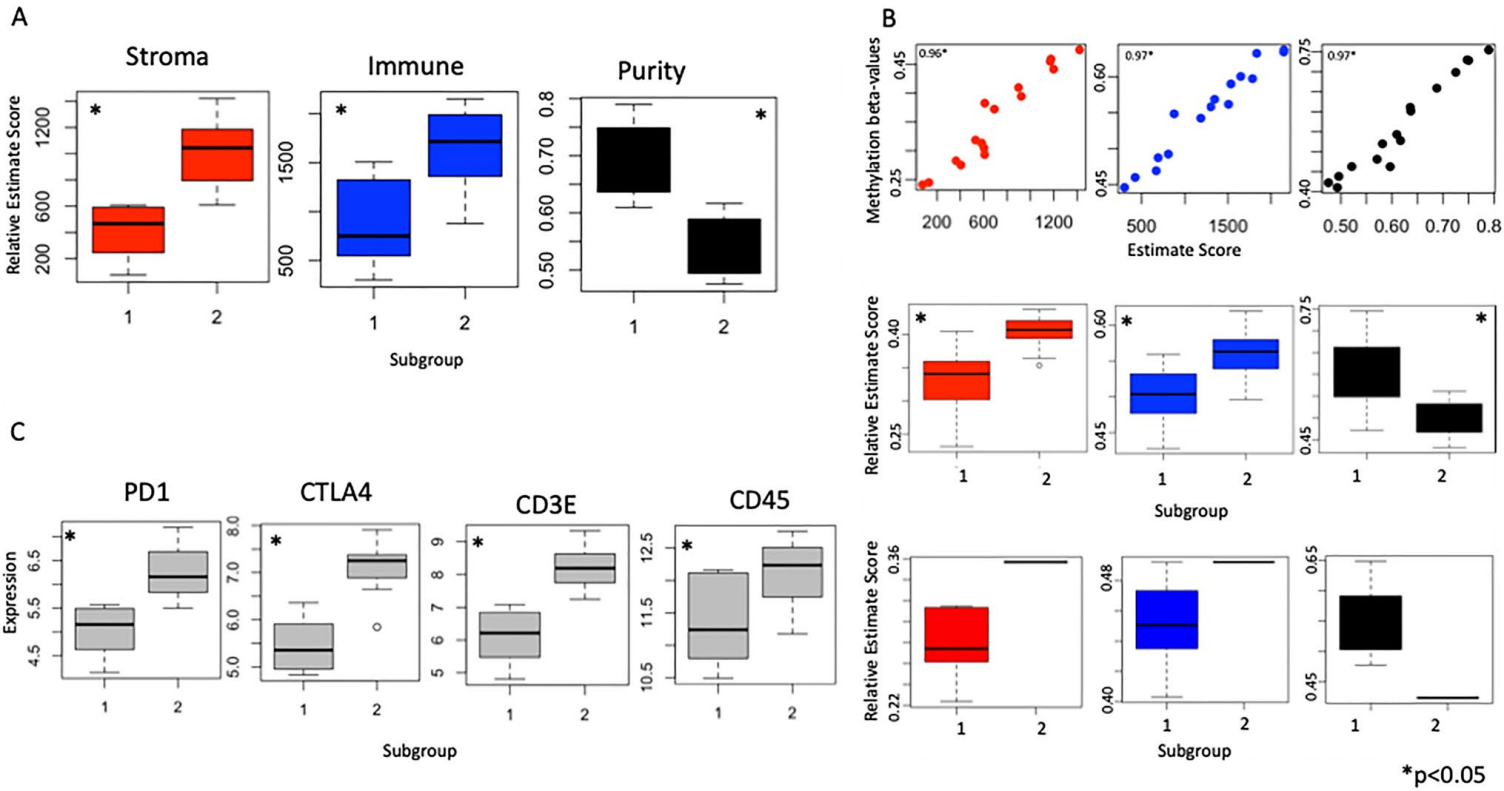
Molecular subgroups exhibit significant differences in microenvironment. **A**: Boxplots comparing relative stroma, immune, and neoplastic (purity) scores between subgroups using the ESTIMATE algorithm. **B**: Validation of key tumour microenvironment (TME) differences by subgroup. Correlation between ESTIMATE signature and the average expression of the 10 most correlated methylation probes in the discovery cohort (stroma, immune, purity from left to right; top row). Pearson correlation values are noted. Boxplots depicting average expression of these 10 methylation probes in the validation cohort 1 (middle row) and 2 (bottom row). **C**: Differential expression of key/targetable immune markers between subgroups in validation cohort

We next applied the ESTIMATE tool to expression data from the discovery cohort to characterize subgroup-specific differences in tumour microenvironment (Fig. 3 A). We find that group 2 has a significantly (p < 0.05) higher proportion of immune and stromal cells, whereas group 1 is enriched in tumour cells. To compare tumour microenvironment in the validation cohorts we developed “methylation signatures” of stroma, immune, and purity scores by taking the average of the ten most tightly correlated methylation probes to each output in the discovery cohort (see methods). Comparing expression of these methylation signatures in the validation cohort revealed similar patterns (Fig. 3B). Similarly, we find that the LUMP score is higher in group 1 for all cohorts, and statistical significance (p < 0.05) is achieved for the discovery and internal validation cohorts.

Finally, differential gene expression analysis revealed 3898 differentially expressed genes (t-test p < 0.05) between subgroups in the discovery cohort. This includes key, and potentially targetable, immune-related genes including current targets of immune checkpoint blockade PD1 and CTLA4, T-cell markers including CD3E, and pan-immune marker CD45 (Fig. 3 C).

### Gene co-expression analysis

**Fig. 4:**
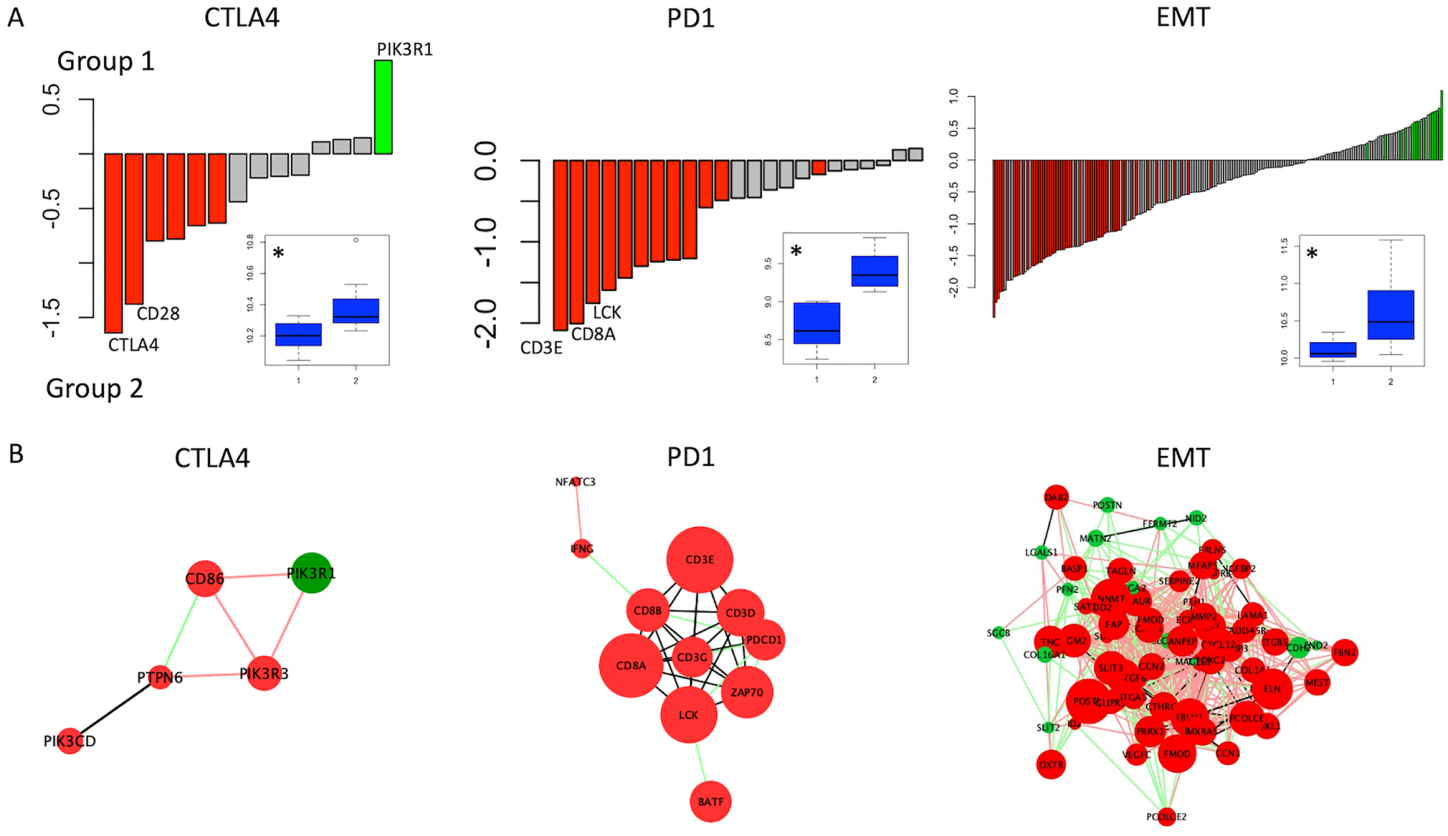
Comparative gene expression analysis. **A**: Differential expression analysis of PD1, CTLA4, and EMT gene networks between subgroups. Mean difference in expression is plotted. Genes significantly upregulated in group 1 (p < 0.05) are coloured green, and those significantly upregulated in group 2 are coloured red. Inset: boxplot comparison of mean expression of genes by group (*p < 0.05). **B**: Gene network analysis. Nodes represent genes whose expression is significantly different between subgroups. Colour corresponds to direction (red is upregulated in group 2, green upregulated in group 1) and size proportional to magnitude of difference in average expression. Edges connect nodes with significant (p < 0.05) co-expression in at least one subgroup; green edges represent connections present only in group 1, red edges represent connections only present in group 2, and black edges represent connections present in both groups. Isolated nodes are not included

Given the subgroup-specific differences in tumour microenvironment identified above, we sought to further characterize related gene networks (identified using *GSEA)* which may form the basis of therapeutic vulnerabilities. We examined PD1 and CTLA4 given their current role in immunotherapy as well as the epithelial to mesenchymal transition (EMT) signature, a critical stroma-associated driver of tumour progression in solid tumours [25],[26]. The PD1 signature is comprised of 23 genes, of which 12 are differentially expressed between subgroups (all upregulated in group 2). The CLTA-4 signature is comprised of 14 genes, of which 7 are differentially expressed between subgroups (1 upregulated in group 1 and 6 upregulated in group 2). Finally, the EMT signature is comprised of 200 genes, of which 71 are differentially expressed between subgroups (15 upregulated in group 1 and 56 upregulated in group 2). Differentially expressed genes within each signature were represented as networks to better understand their subgroup-specific co-expression (Fig. 4). This reinforces the immune- and stroma-enriched nature of group 2 tumours, which we will henceforth refer to as the “immunogenic” subgroup.

### Drug repurposing analysis

We applied the top 100 differentially expressed genes in each direction (up- and down-regulated) for both subgroups. Importantly, MEK/MAPK pathway was present repeatedly as a potential target for group 1 tumours with trametinib as well as experimental agents BRD-K12244279 and PD-98,059 listed as candidate agents. This network consists of 40 genes of which 7 are differentially expressed between subgroups (5 upregulated in group 1 and 2 upregulated in group 2). Given the role of MEK/MAPK in cell cycling, we also examined the *REACTOME* cell cycle signature which contains 693 genes, of which 107 were differentially expressed (70 upregulated in group 1 and 37 upregulated in group 2) [Supplemental Data]. Given these findings, we label group 1 as the “proliferative” subgroup. Other candidate agents for group 1 included vorinostat (a histone deacetylase inhibitor) and tivozanib (a vascular endothelial growth factor receptor inhibitor), whereas candidate agents for group 2 included valrubicin (a topoisomerase inhibitor) and canertinib (an EGFR inhibitor) [Supplemental Data]. Notably, all top 10 candidates in group 2 had different mechanisms of action, unlike in group 1 where multiple MEK inhibitors were present.

## Discussion

### Overview of results

Using a discovery cohort of 16 tumours with both methylation and expression data as well as two validation cohorts with methylation data alone, we find two robust molecular subgroups of vestibular schwannoma. In the discovery cohort, group 1 consists of a higher proportion of neoplastic cells whereas group 2 is enriched in immune and stromal microenvironment. This pattern is consistent in two the validation cohorts. Notably, subgroup assignment is based solely on a methylation signature of 100 differentially methylated probes. No patients in group 1 experienced postoperative recurrence/progression or required subsequent treatment out of four with documented follow up, and 2/6 in group 2 did. Gene co-expression analysis reveals important differences in PD1, CTLA4, EMT, MEK, and cell cycle signalling pathways which suggest important subgroup-specific therapeutic vulnerabilities. We therefore label group 1 as “proliferative” and group 2 as “immunogenic” and suggest the need for subgroup-specific therapies targeted at their unique biology.

### Tumour microenvironment in vestibular schwannoma

Tumour microenvironment has become a topical area of study in oncology and has generated considerable promise for therapeutic considerations in many cancers. Once considered a simple mass of mitotically active cells, we now understand that tumours are complex ecosystems of interacting neoplastic and non-neoplastic cells. These interactions have been shown to stimulate clonal evolution, tumour heterogeneity, immune escape, and treatment resistance, making them a promising target for drug therapy [27]. Perhaps the most poignant example of the tangible results achieved from this understanding is in the effectiveness of immune checkpoint inhibition in cancers such as melanoma [28],[29]. In the CNS, glioblastoma multiforme (GBM) has also been shown to exhibit a region-specific inflammatory microenvironment [30] though PD-1 blockade did not improve overall survival in recurrent GBM when compared to standard of care bevacizumab [31]. This is felt to be secondary, at least in part, to the highly heterogeneous nature of this tumour and ongoing investigation into immunotherapeutics in GBM is underway.

The microenvironment of vestibular schwannoma remains relatively unexplored. However, it has been shown that an increasingly inflammatory microenvironment is associated with higher rates of progression. In a recent review paper [7], increasing proportion of immune cells (identified with surface markers including CD45 and CD68), secretion of inflammatory cytokines (including IL-1, IL-6, TNF), and activation of key regulatory signaling networks such as NF-kB have been associated with tumour proliferation. Interestingly, this microenvironment may be driven in part by systemic inflammation, as one study finds an association between serum C-reactive protein levels and progression-free survival in VS [32]. Immunohistochemical analysis also revealed increased expression of several pro-inflammatory cytokines including tumour necrosis factor, IL-1, IL-6 [33], and CXCR4 [34] in vestibular schwannoma compared to a normal vestibular nerve, suggesting that perhaps the subgroups in our study represent tumours at different points along a spectrum from “normal nerve” to “aggressive tumour”. This is suggested by the fact that subgroup separation in the validation cohorts is not as well defined as in the discovery cohort. It is therefore possible that some tumours fall between the two subgroups and may benefit from multi-pronged approaches to therapy. Larger studies are needed to ascertain the true distribution of tumours between subgroups and the spectrum of cases that lies between them.

While investigation into immunotherapy for vestibular schwannoma has generated some promise, there remains a lack of output. This may be related to the fact that these heterogeneous tumours are still considered a single entity; subgroup-informed analysis of vestibular schwannoma may represent the next frontier of treatment/immunotherapy for this tumour.

### Limitations

A few limitations must be considered in this study. First, the retrospective nature of our study and small numbers may limit generalizability. A lack of available clinical annotation limited our ability to incorporate important variables such as clinical and treatment details in subgroup discovery and analysis, and therefore conclusions regarding clinical behaviour of these subgroups cannot be drawn. The limited number of patients with *SH3PDX2A-HTRA1* fusion in this cohort precluded an analysis of this important mutation, and further work with larger cohorts will be required to determine its relevance in the context of these newly described subgroups. Similarly, the requirement for surgical intervention in obtaining molecular data from these tumours excludes patients treated with radiotherapy or surveillance. Given the often-delayed time to postoperative progression in vestibular schwannoma, it is possible that follow up was insufficient to capture all cases. The young age of the DKFZ cohort confounds direct comparisons, particularly with the lack of clinical and genetic annotation. Finally, lack of expression data in the validation cohort forced indirect comparisons which may have misinformed subgroup generalizability, and precluded validation of subgroup-specific transcriptomic states. Nevertheless, our goal in this work was to identify molecularly relevant subgroups of VS with available data which may represent distinct therapeutic vulnerabilities and/or clinical properties.

### Future directions

The hypotheses generated in this work will serve to inform subsequent prospective studies on larger cohorts with deep multi-omic profiling, allowing validation of our subgroups and further refining their biological landscapes to identify key markers/drivers, which could be verified with immunohistochemistry, and therapeutic vulnerabilities. This will be required to ultimately incorporate our subgroups into clinical practice. Importantly, our subgroups are currently defined based on a signature consisting of only 100 differentially methylated probes. In an era of increasing reliance on whole methylome classification models, this will allow for easy tumour subclassification for preclinical/clinical trial design and ultimately the development of subgroup-specific targeted therapies.

## Conclusions

We leverage established computational tools with multi-omic molecular data to define two robust subgroups of vestibular schwannoma with differences in microenvironment and therapeutic vulnerabilities. While further confirmatory work is required, this promises to increasingly individualize the care of patients with this disease.

## Electronic supplementary material

Below is the link to the electronic supplementary material.

Supplementary Material 1
