# Supplementary material for: Multiplatform molecular analysis of vestibular schwannoma reveals two robust subgroups with distinct microenvironment
Source: J Neurooncol. 2023 Jan 26;161(3):491–9. doi: 10.1007/s11060-022-04221-2 (PMC9992225; doi:10.1007/s11060-022-04221-2)
Supplement: Supplementary file 1 — Supplementary Material 1 [file 11060_2022_4221_MOESM1_ESM.pdf]

Drug	MOA	Connectivity score
UNC-0321	Histone lysine methyltransferase inhibitor	1.84
BRD-K12244279	MEK inhibitor	1.77
Tivozanib	VEGFR inhibitor	1.76
Trametinib	MEK inhibitor	1.75
PD-98059	MEK/MAPK inhibitor	1.75
E-2012	Gamma secretase inhibitor	1.74
Voronistat	HDAC inhibitor	1.72
Felodipine	Calcium channel blocker	1.71
SB-705798	TRPV antagonist	1.71
Telmisartan	Angiotensin receptor antagonist	1.69

Supplemental Table 1: Top repurposed drug candidates for treatment of group 1 tumours as identified by the L1000 drug repurposing hub (input is inverse of previous). Top 100 up- and down- regulated genes are used as input. Top 10 candidate drugs are shown (red represents entities that have shown promise in other cancers).

Drug	MOA	Connectivity score
UNC-0638	Histone methyltransferase inhibitor	1.98
BRD-K61734568	BCL inhibitor	1.81
P-5091	Ubiquitin-specific protease inhibitor	1.81
Methylnaltrexone	Opioid receptor antagonist	1.80
VLX-600	USP-14 inhibitor	1.78
QL-XII-47	BTK inhibitor	1.75
Valrubicin	Topoisomerase inhibitor	1.74
Digoxin	Na-K ATPase inhibitor	1.73
Cefoperazone	Bacterial cell wall synthesis inhibitor	1.73
Canertinib	EGFR inhibitor	1.73

Supplemental Table 2: Top repurposed drug candidates for treatment of group 2 tumours as identified by the L1000 drug repurposing hub. Top 100 up- and down- regulated genes are used as input. Top 10 candidate drugs are shown (red represents entities that have shown promise in other cancers).

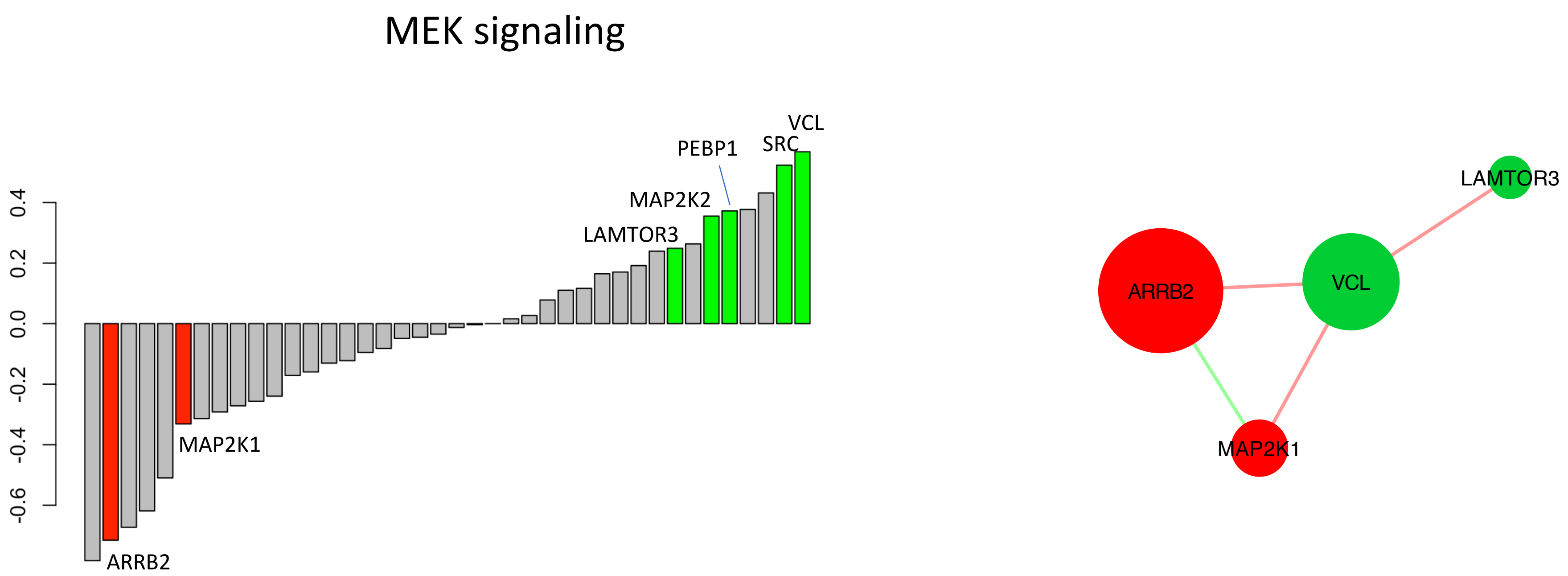

Supplemental Figure 1: Gene network analysis of MEK signaling. Conventions are the same as those used in Figure 4.

Cell cycle genes

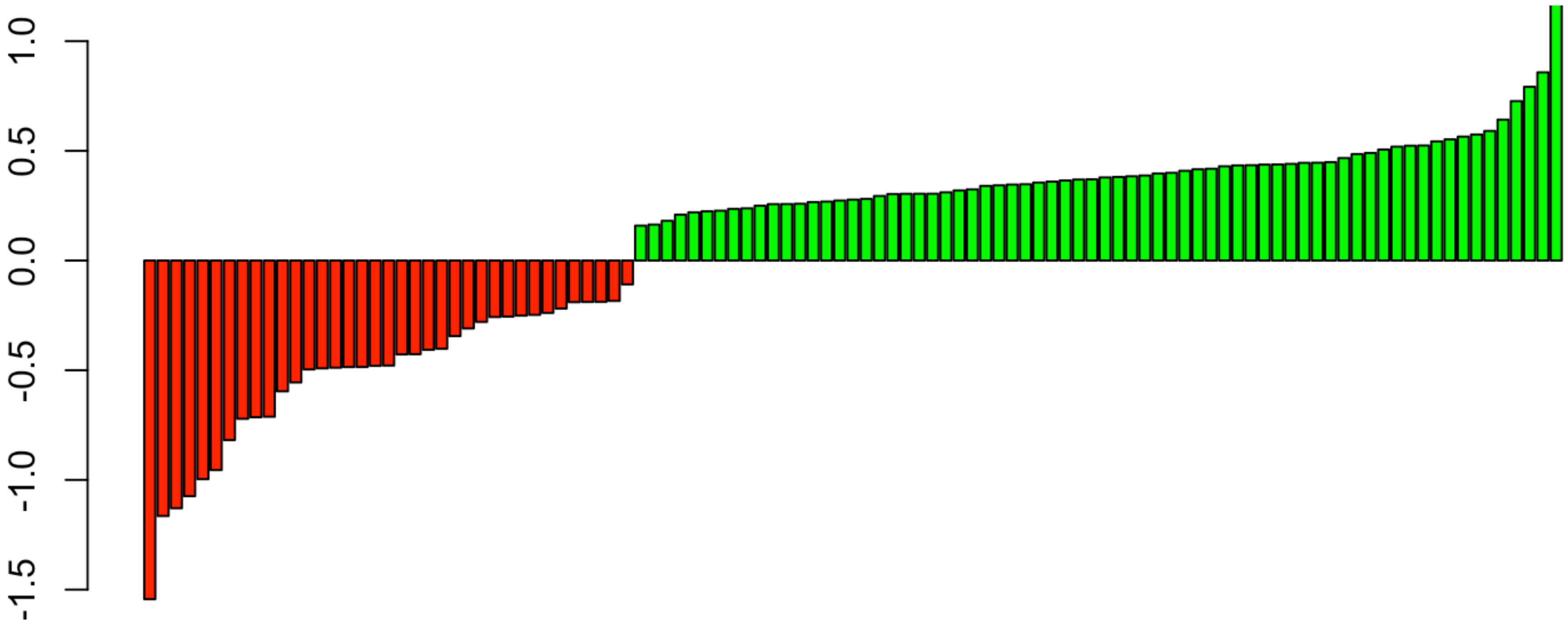

Supplemental Figure 2: Gene network analysis of cell cycle. Only significantly different genes plotted given number of genes. Cytoscape plot not included due to size.
